# An open-label multi-center phase 1 safety study of BXQ-350 in children and young adults with relapsed solid tumors, including recurrent malignant brain tumors

**DOI:** 10.1016/j.heliyon.2022.e12450

**Published:** 2022-12-19

**Authors:** Mohamed S. Abdelbaki, Mariko Dawn DeWire Schottmiller, Timothy P. Cripe, Richard C. Curry, Charles A. Cruze, Leah Her, Suzanne Demko, Denise Casey, Bhuvana Setty

**Affiliations:** a Washington University School of Medicine, Department of Pediatrics, St. Louis, MO, United States; b Medpace, Cincinnati, OH, United States; c Nationwide Children’s Hospital, Columbus, OH, United States; d CTI Clinical Trials and Consulting, Covington, KY, United States; e Bexion Pharmaceuticals, Cincinnati, OH, United States; f DataRevive, Rockville, MD, United States

**Keywords:** Pediatric oncology, Tumors, Brain, Phase I clinical Trials, Rare tumors

## Abstract

**Background:**

BXQ-350 is a novel anti-neoplastic agent composed of saposin C (SapC) and phospholipid dioleoylphosphatidyl-serine sodium (DOPS) that selectively binds tumor cell phosphatidylserine (PS), inducing apoptosis. BXQ-350 has demonstrated preclinical antitumor effects in high-grade gliomas (HGG) and clinical activity in adult patients with recurrent HGG.

**Methods:**

A phase 1 study was conducted in pediatric patients with relapsed/refractory solid tumors, including recurrent brain tumors. Primary objectives were to characterize safety and determine maximum tolerated dose (MTD) and preliminary antitumor activity. Sequential dose cohorts were assessed up to 3.2 mg/kg using an accelerated titration design. Each cycle was 28 days; dosing occurred on days 1–5, 8, 10, 12, 15, and 22 of cycle 1, and day 1 of subsequent cycles, until disease progression or toxicity.

**Results:**

Nine patients, median age 10 years (range: 4–23), were enrolled. Seven patients (78%) had central nervous system (CNS) and two (22%) had non-CNS tumors. Eight patients completed cycle 1. No dose limiting toxicity (DLT) or BXQ-350-related serious adverse events (SAEs) were observed. Six patients experienced at least one adverse event (AE) considered possibly BXQ-350-related, most were grade ≤2. One patient with diffuse intrinsic pontine glioma experienced stable disease for 5 cycles. The study was terminated after part 1 to focus development on the frontline setting.

**Conclusion:**

No DLTs or BXQ-350-related SAEs were reported, and the maximal planned dose of 3.2 mg/kg IV was tolerable. Limited safety and efficacy data support continued BXQ-350 development in pediatric HGG; however, early discontinuations for progression suggest novel therapies be assessed at earlier disease stages.

## Highlights

• BXQ-350 is a novel drug utilizing sphingolipid metabolism.
• Phase 1 dose escalation study of BXQ-350 in pediatric solid tumors including CNS.
• BXQ-350 is a well-tolerated intravenous drug with a Maximum Planned Dose of 3.2 mg/kg.

## 1 Introduction

Central nervous system (CNS) tumors are the second most common type of childhood cancer and the leading cause of childhood cancer deaths in the United States (U.S.) [1]. The average annual age-adjusted incidence is 6.06 per 100,000 U.S. population [2]. Five-year survival for pediatric patients with CNS tumors (75%) is lower than that for pediatric cancers overall (86%) [1]. High-grade glioma (HGG), representing 30% of all pediatric gliomas, and diffuse intrinsic pontine glioma (DIPG), the most common brainstem cancer, have 5-year survival rates less than 20% and 1%, respectively [3].

Current standard treatment options for pediatric CNS tumors consist of surgery, radiation, and/or chemotherapy [3]. Surgical resection is typically not an option for patients with DIPG because of location and infiltrative growth pattern [3, 4]. Chemotherapy and radiation therapy are associated with long-term adverse neurological and neuroendocrine outcomes [5]. Although several targeted therapies are under investigation in clinical trials, none have been approved for pediatric DIPG or HGG [4]. There remains an unmet medical need for novel therapeutic agents with improved efficacy and tolerability.

BXQ-350 is a novel anti-neoplastic agent comprised of saposin C (SapC), an expressed human lysosomal protein, and the phospholipid dioleoylphosphatidyl-serine sodium (DOPS), a phospholipid that has a similar composition and structure to those of phosphatidylserine located on cell membranes. In the tumor microenvironment, BXQ-350 selectively targets and binds phosphatidylserine (PS), which is expressed at high levels on the outer leaflet of the plasma membrane in tumor cells [6, 8, 9, 10, 11, 12]. SapC is a known allosteric activator of multiple enzymes controlling sphingolipid metabolism, causing the production and accumulation of ceramide, leading to cell death [6, 13]. Preclinical studies have demonstrated that BXQ-350 can cross the blood brain barrier, target tumor tissue and induce cell death in several different cancer types, including primary and metastatic brain tumors [6, 7, 11, 14]. It also acts synergistically with radiation and several chemotherapeutic agents.

Results from the first in human adult phase 1 study of BXQ-350 in advanced solid tumors and recurrent HGG (NCT02859857) demonstrated that BXQ-350 was well-tolerated at doses up to the planned maximum dose level of 2.4 mg/kg. No maximum tolerated dose (MTD) was reached and 2.4 mg/kg intravenous (IV) every 4 weeks was determined to be safe in adults with solid tumors, including HGG. Two patients experienced a partial response and multiple patients had prolonged stable disease (eight up to 6 months, seven up to 12 months, and two up to 24 months). In addition, five patients at 2.4 mg/kg remaining on BXQ-350 were enrolled in a continued treatment study (NCT04404569); two of whom remain on BXQ-350 over 6 years as of November 2022. Several patients that experienced a clinical benefit included GBM and CNS patients suggesting that BXQ-350 distributed in the brain and CNS compartments at clinically effective concentrations These results provided rationale for development in the pediatric population with advanced CNS tumors. A two-part, open-label, multi-center, phase 1 dose escalation study of BXQ-350 in pediatric patients with relapsed solid tumors, including recurrent malignant brain tumors (NCT03967093), was conducted between April 15, 2019 and January 3, 2020. The primary objectives were to characterize the safety profile and determine the MTD of BXQ-350 (part 1) and to assess the preliminary antitumor activity of BXQ-350 (part 2).

## 2 Methods

### 2.1 Patient eligibility criteria

Eligible patients were age ≥1–30 years with histologically or cytologically confirmed relapsed solid tumors, including recurrent malignant CNS solid tumors, with no available treatment options. Patients were required to have a Lansky or Karnofsky performance score of >50 or Eastern Cooperative Oncology Group performance status (ECOG PS) of ≤2. Adequate liver, renal, coagulation, and bone marrow function were also required. Patients with concurrent or second malignancy, lymphoma, Grade ≤2 glioma, Grade 1 ependymoma, symptomatic brain metastases, or leptomeningeal disease were excluded. Patients who received anti-cancer therapies within 2–6 weeks, myelosuppressive agents within 3–4 weeks, monoclonal antibodies within 4 weeks, growth factors or immunotherapy within 2–4 weeks, or any investigational drug or major surgery within 4 weeks of the first study dose, were excluded. Patients must have recovered from toxicities of prior therapies or any surgical procedure before enrollment.

Institutional review board (IRB) approval was obtained at Nationwide Children’s Hospital (Approval Number: STUDY0000019) and Cincinnati Children’s Hospital Medical Center (Approval Number: 2019-0489).

A written consent was obtained from each patient or guardian. An assent was obtained, as appropriate, according to the institution and IRB standards.

### 2.2 Drug administration and study design

BXQ-350 was supplied as lyophilized white powder in a single-use glass vial containing 8.8 mg of SapC. BXQ350 was reconstituted with sterile water and diluted in normal saline. After preparation, BXQ-350 was stable at 2–8 °C for up to 48 h or 15–30 °C for up to 24 h. All dosing calculations were based on the content of SapC per vial and patient weight.

Premedication with an antihistamine was considered optional for the first 2 patients; however, premedication was required for all patients after June 14, 2019 (protocol amendment v8.0) based on a Grade 4 infusion reaction observed in a single patient in part 3 of the adult phase 1 trial. Patients were allowed concomitant analgesics, antiemetics, antibiotics, antipyretics, and blood products as needed. All concomitant medications were recorded in the case report form.

Treatment cycles were based on experience in the adult study and were 28 days in length. BXQ-350 was administered as an intravenous infusion on days 1–5, 8, 10, 12, 15, and 22 during cycle 1, and day 1 of subsequent cycles, for a maximum of 6 cycles, until disease progression, or unacceptable toxicity.

Dose-escalation in part 1 occurred using a standard 3 + 3 design following an accelerated dose titration via single patient cohorts for the first 2 dose levels. The starting dose was 1.8 mg/kg (n = 1), and dose escalation occurred at 30% increments to 2.4 mg/kg (n = 1) and 3.2 mg/kg (n = 7). The maximum single dose administered to any patient was limited to 294 mg total to account for extremely obese patients. A safety monitoring committee reviewed all safety data from each cohort prior to proceeding with dose escalation. The planned dose expansion for part 2 at 3.2 mg/kg IV on days 1–5, 8, 10, 12, 15, and 22 during cycle 1, and day 1 of subsequent cycles, was approved by the safety committee; however, the study was terminated following part 1. The sponsor elected to terminate the study, after establishing the Part 2 dose, and to pursue the development of BXQ-350 as an earlier treatment measure for the pediatric orphan drug indication of DIPG/DMG.

Adverse events (AEs) were graded per the National Cancer Institute (NCI) Common Terminology Criteria for Adverse Events (CTCAE) version 5.0. Dose limiting toxicities (DLTs) were defined as BXQ-350-related hematologic toxicity of Grade 4 neutropenia lasting >7 days, Grade 4 thrombocytopenia, Grade 3 thrombocytopenia with clinically significant bleeding or requiring a platelet transfusion, Grade 4 anemia, and/or any Grade ≥3 non-hematologic toxicity (excluding alopecia, lymphopenia, and Grade 3 nausea, vomiting, diarrhea, and electrolyte imbalances lasting less than 48 h).

### 2.3 Study assessments

Study assessments included medical history, prior/concomitant medications, disease assessment, physical exam, vital signs, electrocardiogram, age-specific performance scores [Lansky (age 1–15), Karnofsky (age ≥16) or ECOG PS (age ≥18)], hematology, chemistry, coagulation, and urinalysis. Patients were assessed for AEs at each study visit. Laboratory safety assessments were conducted on days 1 and 22 for coagulation, and weekly during cycle 1 and day 1 of each subsequent cycle for hematology, chemistry, and urinalysis. Tumor response was evaluated 4 and 8 weeks after the first treatment, then every 8 weeks thereafter for the duration of treatment using the Response Evaluation Criteria in Solid Tumors (RECIST) v1.1 for non-CNS solid tumors or Radiographic Response Criteria (RRC) for recurrent malignant CNS solid tumors. Serum anti-drug antibody (ADA) assessments occurred on days 1, 22, and 113 for development of tests designed to identify patients most likely to respond positively or negatively to BXQ-350 and were analyzed by bridging electrochemiluminescence using a validated method for the detection of antibodies against SapC. Any putative positive samples were confirmed.

## 3 Results

### 3.1 Patient characteristics

Nine patients were enrolled in part 1 of the study between April 15, 2019 and January 3, 2020. Patient characteristics are summarized in Table 1. Seven (78%) patients had CNS tumors and two (22%) had non-CNS tumors.

**Table 1:** Patient characteristics.

Characteristics	BXQ-350 N = 9 n (%)
**Age (years)**	
Median	10
Range	4–23
**Gender**	
Male	5 (56)
Female	4 (44)
**Race**	
Black	2 (22)
Other	1 (11)
White	5 (56)
White, other	1 (11)
**Ethnicity**	
Hispanic or Latino	1 (11)
Not Hispanic or Latino	8 (89)
**Performance status**	
Lansky (age 1–15)	
60	1 (11)
70	2 (22)
80	2 (22)
90	2 (22)
Karnofsky (age ≥16)	
90	1 (11)
ECOG (age ≥18)	
0	1 (11)
**Diagnosis**	
Brain neoplasm malignant^1^	4 (44)
Ependymoma malignant	1 (11)
Glioblastoma	1 (11)
Osteosarcoma recurrent	2 (22)
Pineoblastoma	1 (11)

1 Two patients with pontine gliomas; one patient with brain neoplasm in the right thalamus/temporal lobe; one patient with brain neoplasm in the brainstem/thalamus.

Prior cancer therapies or surgeries are outlined in Table 2.

**Table 2:** Prior cancer therapy in study participants.

Preferred Term	BXQ-350 N = 9 n (%)
**Surgical procedures**	8 (89)
Tumor excision	3 (33)
**Radiation therapy**	7 (78)
Radiotherapy to brain	6 (66)
Radiotherapy to bone	1 (11)
**Systemic therapy**	9 (100)
Antineoplastic agents	9 (100)
Bevacizumab	2 (22)
Cisplatin	3 (33)
Cyclophosphamide	2 (22)
Doxorubicin	2 (22)
Etoposide	4 (44)
Everolimus	2 (22)
Gemcitabine	2 (22)
Ifosfamide	2 (22)
Investigational antineoplastic drugs	3 (33)
Irinotecan	2 (22)
Paclitaxel albumin	2 (22)
Ribociclib	2 (22)
Temozolomide	3 (33)
Immunosuppressants	3 (33)
Methotrexate	3 (33)

All patients underwent prior therapy including tumor excision (33%), radiation (78%) and systemic chemotherapy (100%). The most common prior systemic treatments (at least three patients) were etoposide (44%), cisplatin (33%), temozolomide (33%), methotrexate (33%), and investigational antineoplastic drugs (33%).

Eight of the nine enrolled patients completed at least 1 cycle of BXQ-350, six patients completed at least 2 cycles, and one patient completed 5 cycles. One patient in the 3.2 mg/kg cohort withdrew consent during cycle 1 after receiving 6 doses due to grade 4 respiratory failure and hypotension unrelated to BXQ-350. The events were considered secondary to progressive disease and this patient was replaced per protocol.

All patients who received any portion of at least one BXQ-350 infusion were included in the safety analyses. Response to treatment was evaluated as an exploratory analysis in all patients who had measurable disease at screening and at least one on-study disease assessment.

### 3.2 Toxicity assessment

All patients experienced at least one AE. No DLTs occurred in the study. Five patients enrolled in dose levels 2.4 mg/kg (n = 1) and 3.2 mg/kg (n = 4) experienced a total of 19 SAEs, all of which were considered unrelated or unlikely related to BXQ-350. SAEs that occurred in more than one patient were depressed level of consciousness (n = 2), seizure (n = 2), hydrocephalus (n = 2), and respiratory failure (n = 2). One patient was discontinued from BXQ-350 due to respiratory failure and hypotension related to progressive lung disease. No AEs led to dose interruptions or reductions during the study.

Common AEs (≥three patients) are summarized in Table 3. The most frequent AEs occurring in at least patients were sinus bradycardia, constipation, fatigue, gait disturbance, back pain, pain in extremity, headache, hemiparesis, cranial nerve VI disorder, and hypertension.

**Table 3:** Treatment-emergent adverse events in ≥3 patients across all cohorts.

	BXQ-350 1.8 mg/kg N = 1 n (%)		BXQ-350 2.4 mg/kg N = 1 n (%)		BXQ-350 3.2 mg/kg N = 7 n (%)	
	All Grades	Grades ≥3	All Grades	Grades ≥3	All Grades	Grades ≥3
**Cardiac disorder**						
Sinus bradycardia	0 (0)	0 (0)	1 (100)	0 (0)	3 (43)	1 (14)
**Endocrine disorders**						
Cushingoid	0 (0)	0 (0)	0 (0)	0 (0)	3 (43)	0 (0)
**Eye disorders**						
Eye pain	1 (100)	0 (0)	1 (100)	0 (0)	1 (14)	0 (0)
**Gastrointestinal disorders**						
Constipation	1 (100)	0 (0)	1 (100)	0 (0)	4 (57)	1 (14)
Dysphagia	1 (100)	0 (0)	0 (0)	0 (0)	3 (43)	2 (29)
Nausea	0 (0)	0 (0)	0 (0)	0 (0)	3 (43)	0 (0)
Vomiting	0 (0)	0 (0)	0 (0)	0 (0)	3 (43)	0 (0)
**General disorders and administration site conditions**						
Disease progression^1^	0 (0)	0 (0)	1 (100)	1 (100)	2 (28.6)	2 (28.6)
Fatigue	1 (100)	0 (0)	1 (100)	1 (100)	4 (57)	2 (29)
Gait disturbance	1 (100)	0 (0)	1 (100)	1 (100)	2 (29)	2 (29)
**Investigations**						
Weight increased	0 (0)	0 (0)	1 (100)	0 (0)	2 (29)	0 (0)
**Metabolism and nutrition disorders**						
Appetite decreased	0 (0)	0 (0)	1 (100)	0 (0)	2 (29)	1 (14)
Hyperglycemia	1 (100)	0 (0)	1 (100)	0 (0)	1 (14)	0 (0)
**Musculoskeletal and connective tissue disorders**						
Back pain	0 (0)	0 (0)	1 (100)	0 (0)	3 (43)	0 (0)
Pain in extremity	0 (0)	0 (0)	0 (0)	0 (0)	4 (57)	1 (14)
**Nervous system disorders**						
Depressed level of consciousness	0 (0)	0 (0)	1 (100)	0 (0)	2 (29)	1 (14)
Dizziness	0 (0)	0 (0)	1 (100)	1 (100)	2 (29)	0 (0)
Facial nerve disorder	0 (0)	0 (0)	0 (0)	0 (0)	3 (43)	0 (0)
Headache	0 (0)	0 (0)	1 (100)	0 (0)	4 (57)	1 (14)
Hemiparesis	0 (0)	0 (0)	1 (100)	0 (0)	3 (43)	3 (43)
Hydrocephalus	0 (0)	0 (0)	1 (100)	1 (100)	2 (29)	1 (14)
VI^th^ nerve disorder	1 (100)	0 (0)	1 (100)	0 (0)	2 (29)	0 (0)
**Psychiatric disorders**						
Irritability	1 (100)	0 (0)	0 (0)	0 (0)	2 (29)	0 (0)
**Respiratory, thoracic, and mediastinal disorders**						
Cough	1 (100)	0 (0)	0 (0)	0 (0)	2 (29)	0 (0)
**Skin and subcutaneous tissue disorders**						
Alopecia	0 (0)	0 (0)	0 (0)	0 (0)	3 (43)	0 (0)
**Vascular disorders**						
Hypertension	0 (0)	0 (0)	1 (100)	1 (100)	4 (57)	3 (43)
Hypotension	1 (100)	0 (0)	0 (0)	0 (0)	2 (29)	1 (14)

AEs included both serious and non-serious.

Any patients with multiple events in one SOC was counted only once for that SOC, using the event with the greatest severity. Any patient with multiple events in one PT was counted only once for that PT, using the event with the greatest severity.

1 Included 3 patients with fatal disease progression.

Six patients (67%) enrolled in dose levels 1.8 mg/kg (n = 1) and 3.2 mg/kg (n = 5) had 11 AEs reported as “possibly related” including nausea, vomiting, fatigue, pneumonia, hypertension, proteinuria, and blood creatinine increased (Table 4). These AEs were grade 1 or 2 in severity except for one grade 3 pneumonia. Six of the eleven AEs resolved (including 2 events that recovered with sequelae), four AEs were considered not resolved (2 patients with Grade 1 fatigue and one patient with Grade 1 proteinuria and Grade 3 pneumonia who was replaced early on study), and 1 AE (Grade 2 nausea) was on-going at the time of death.

**Table 4:** Treatment-related adverse events related to BXQ-350.

	BXQ-350 1.8 mg/kg N = 1 n (%)		BXQ-350 2.4 mg/kg N = 1 n (%)		BXQ-350 3.2 mg/kg N = 7 n (%)	
	All Grades	Grades ≥3	All Grades	Grades ≥3	All Grades	Grades ≥3
**Gastrointestinal disorders**						
Nausea	0 (0)	0 (0)	0 (0)	0 (0)	2 (29)	0 (0)
Vomiting	0 (0)	0 (0)	0 (0)	0 (0)	1 (14)	0 (0)
**Infections and Infestations**						
Pneumonia	0 (0)	0 (0)	0 (0)	0 (0)	1 (14)	1 (14)
**Investigations**						
Blood creatinine increased	0 (0)	0 (0)	0 (0)	0 (0)	1 (14)	0 (0)
**General disorders and administration site conditions**						
Fatigue	1 (100)	0 (0)	0 (0)	0 (0)	2 (29)	0 (0)
**Renal and urinary disorders**						
Proteinuria	0 (0)	0 (0)	0 (0)	0 (0)	1 (14)	0 (0)
**Vascular disorders**						
Hypertension	0 (0)	0 (0)	0 (0)	0 (0)	1 (14)	0 (0)

No patient died on study due to an AE. Three patients (33%) at doses of 2.4 mg/kg (n = 1) and 3.2 mg/kg (n = 2) had disease progression recorded as fatal events. Two were discontinued from study due to disease progression prior to their deaths; one death occurred while on study due to the rapid onset of progression.

### 3.3 Tumor response

Disease assessments were conducted in seven of the nine patients enrolled using RECIST v1.1 for patients with non-CNS tumors and RRC for those with CNS tumors; two patients (osteosarcoma-1 and glioblastoma-1) discontinued treatment prior to the scheduled day 29 disease assessment. Five patients (71%; 1 non-CNS and 4 CNS) had stable disease (one patient each at 1.8 mg/kg and 2.4 mg/kg, and three patients at 3.2 mg/kg) and two patients with CNS disease had evidence of progression on day 29. One patient with DIPG (3.2 mg/kg) was initially observed on day 29 with suspected pseudo-progression but was confirmed to be stable via brain magnetic resonance imaging scan. This patient continued to have stable disease on day 113 and remained on study until day 131.

### 3.4 Anti-drug antibodies

Nineteen samples were collected from nine patients on day 1, 8 patients on day 22, one patient on day 113, and one patient at end of study treatment, for ADA assessment. All collected samples analyzed were negative for anti-SapC.

## 4 Discussion

This was the first pediatric study conducted with BXQ-350. The dose selection was based on the severely toxic dose 10 (STD10) of 40 mg/kg and the highest non-severely toxic dose (HNSTD) of 20 mg/kg determined from rat and monkey toxicology studies, respectively, and on experience in the adult phase 1 trial of BXQ-350. The proposed starting dose of 1.8 mg/kg represented a 30% reduction of the highest target dose level of 2.4 mg/kg achieved in the adult clinical study. The dose administration schedule mirrored the adult phase 1 trial.

Overall, the study results indicate that the planned maximum dose of 3.2 mg/kg is safe and tolerable in pediatric patients. AEs observed in the study were generally reflective of the patient population with advanced cancers and progressive disease. No infusion related reactions were observed. At the time of enrollment, all patients had received at least one (or combination of) prior surgery, radiation and/or systemic therapy. No MTD or recommended Phase 2 dose (RP2D) was determined, and no DLTs or BXQ-350-related SAEs were observed; safety findings were consistent with the reported results of the phase 1 BXQ-350 study in adults [15]. All patients ultimately experienced progressive disease and subsequently discontinued from study, died, or withdrew consent.

Although one patient received 5 cycles of BXQ-350, most patients progressed and discontinued prior to cycle 3, suggesting that the population studied had disease too advanced to complete a 6-cycle regimen. The planned dose expansion in part 2 at 3.2 mg/kg was approved by the safety committee; however, the study was terminated following part 1. Considering the encouraging safety and efficacy results from the adult clinical study (NCT02859857), (manuscript in preparation) in which signs of clinical benefits were observed in recurrent GBM patients (1 patient had a partial response and remained on study for 16 months; 1 patient with stable disease on study for over 6 years) and other CNS tumors (three recurrent ependymoma patients had stable disease for up to 6 months), and the good safety profile of BXQ-350 in the pediatric population suggest that BXQ-350 could be combined with standard of care in different indications, including GBM and CNS tumors. As a result, resources shifted to focus on BXQ-350 development as part of front-line treatment for high grade pediatric brain tumors with standard of care, a population for which where there remains an unmet need for new therapies that may offer curative potential and where higher BXQ-350 doses could be explored.

There were limitations to this study. The study design provided a small sample size, and there was lack of pharmacokinetic (PK) data available to support the pediatric recommended dose regimen. PK sampling was planned for part 2 of the study but did not occur due to study termination. PK/pharmacodynamic (PD) will be assessed in the new pediatric study with DIPG or DMG.

The ability of BXQ-350 to cross the blood brain tumor barrier and target tumor tissue was demonstrated using in vitro and in vivo models [6]. In preclinical models, BXQ-350 was able to selectively target tumor cells to induce cell death in multiple tumor types, including GBM. Additionally, in both in vitro and in vivo models, BXQ-350 acts synergistically with both radiation and chemotherapeutic agents against various target tumor types. In the adult phase 1 trial (NCT02859857), 2 patients (GBM-1) achieved partial responses and 8 patients (GBM-2) were observed with progression-free survival >6 months (range: 6–63 months). Five patients (GBM-2) were subsequently enrolled into a continuation treatment study (NCT04404569). In the pediatric phase 1 study (NCT03967093), one patient with DIPG had stable disease until day 113 and received treatment for 5 cycles, and an acceptable safety profile was demonstrated up to the maximum planned dose level of 3.2 mg/kg across the pediatric study population. Taken together, data suggest that the addition of BXQ-350 early in a treatment regimen in combination with radiation or other chemotherapeutic agent may offer advantages, and further dose escalation may be considered. Pediatric patients with HGG, particularly, DIPG/diffuse midline glioma (DMG) were selected for investigation in the front-line setting and the combination of BXQ-350 and radiation therapy is expected to further enhance tumor cell death and offer an advantage over radiation alone [16]. Preclinical in vitro studies demonstrated that BXQ-350 could induce apoptotic cell death in a DIPG cell line (unpublished) Other studies have suggested that fractionated radiation enhanced the effect of SapC-DOPS in some non-DIPG cancer cell lines [17]. Currently, a pediatric study of BXQ-350 and radiation therapy in patients with newly diagnosed DIPG or DMG patients with H3K27M mutation is open for enrollment (NCT04771897).

## Declarations

### Author contribution statement

Mohamed S Abdelbaki; Mariko Dawn DeWire Schottmiller; Timothy P Cripe; Richard C Curry; Charles A Cruze; Leah Her; Susanne Demko; Bhuvana Setty: Analyzed and interpreted the data; Wrote the paper.

### Funding statement

This research did not receive any specific grant from funding agencies in the public, commercial, or not-for-profit sectors.

### Data availability statement

Data included in article/supp. material/referenced in article.

### Declaration of interest’s statement

The authors declare the following conflict of interests: Dr. Richard Curry has financial stock in Bexion Pharmaceuticals that total <1% of the company.

### Additional information

No additional information is available for this paper.
